# A Global Estimate of Seafood Consumption by Coastal Indigenous Peoples

**DOI:** 10.1371/journal.pone.0166681

**Published:** 2016-12-05

**Authors:** Andrés M. Cisneros-Montemayor, Daniel Pauly, Lauren V. Weatherdon, Yoshitaka Ota

**Affiliations:** 1 NEREUS Program, Institute for the Oceans and Fisheries, The University of British Columbia, Vancouver, Canada; 2 Sea Around Us Project, Institute for the Oceans and Fisheries, The University of British Columbia, Vancouver, Canada; 3 UNEP World Conservation Monitoring Centre, Cambridge, United Kingdom; University of Tasmania, AUSTRALIA

## Abstract

Coastal Indigenous peoples rely on ocean resources and are highly vulnerable to ecosystem and economic change. Their challenges have been observed and recognized at local and regional scales, yet there are no global-scale analyses to inform international policies. We compile available data for over 1,900 coastal Indigenous communities around the world representing 27 million people across 87 countries. Based on available data at local and regional levels, we estimate a total global yearly seafood consumption of 2.1 million (1.5 million–2.8 million) metric tonnes by coastal Indigenous peoples, equal to around 2% of global yearly commercial fisheries catch. Results reflect the crucial role of seafood for these communities; on average, consumption per capita is 15 times higher than non-Indigenous country populations. These findings contribute to an urgently needed sense of scale to coastal Indigenous issues, and will hopefully prompt increased recognition and directed research regarding the marine knowledge and resource needs of Indigenous peoples. Marine resources are crucial to the continued existence of coastal Indigenous peoples, and their needs must be explicitly incorporated into management policies.

## Introduction

Indigenous groups include some 370 million people, 5% of the global population, and an overwhelming number exist in precarious socioeconomic and political conditions [1]. Along the world’s oceans, coastal Indigenous peoples (CIPs) share vital links to marine ecosystems that conserve their cultural heritage and underpin food sovereignty (the right to define and access healthy and culturally-appropriate food [2]). At the same time, these strong links make them particularly vulnerable to challenges—including declines of coastal marine fish populations [3,4], climate change [5], pollution, and multi-scale social-economic dynamics [6,7]—that affect their fishing practices [8–10].

In the context of globalization, exogenous economic and political pressures affect both the ecological setting [11] and socio-cultural practices of Indigenous fisheries worldwide [12]. Poverty, cultural biases, and legal traps already threaten CIPs’ security [13], impacting social organization, public health, and increasing resource related conflict [14]. Reduced interactions with marine ecosystems and access to fish, aside from negative economic impacts, can further exacerbate loss of cultural transmission, language [15] and physical communities [16]. For instance, political displacement linking recognition and rights to a defined spatial area limits access to marine resources and can act as a barrier to self-reliance [17]. Furthermore, although there are concerns due to bioaccumulation of pollutants in seafood [18,19], changes to traditional diets have significant implications for Indigenous peoples with predisposition to chronic health issues such as diabetes and heart disease [20–22].

The critical status of Indigenous peoples and their relation to marine resources in particular have been recognized in a number of international agreements. The UN Declaration on the Rights of Indigenous People [23] addresses the socioeconomic conditions of this sector and the challenges posed by environmental degradation and loss of natural resources. In creating solutions to these issues, particularly those involving marine living resources and biodiversity, the Convention on Biological Diversity [24] recognizes the value of traditional ecological knowledge and the FAO Small-Scale Fisheries Guidelines [25] supports Indigenous forms of governance and preferential access rights. The Intergovernmental Panel on Climate Change (IPCC) has also called for special attention to vulnerable and resource-dependent Indigenous peoples since climate change is expected to exacerbate stakeholder inequalities [26].

Despite this recognition, no quantitative studies exist that provide a sense of scale to coastal Indigenous issues at the global level. This is partly due to research difficulties in what are often small and remote communities (e.g., [27–29]), and a deeper lack of official recognition of the activities and needs of Indigenous peoples in general [30]. Ethnographic research—key for Indigenous issues—has historically focused on the qualitative aspects of these fisheries and on providing essential socially-contextualized perspectives, but less on quantitative data [11,31–33]. However, we argue that advancing the development and implementation of international policies supporting Indigenous peoples urgently require quantitative data on their marine living resource needs across the world [10].

The objective of this study is to provide an initial estimate of the marine coastal Indigenous population and the yearly amount of seafood necessary to meet their consumptive needs. We develop a methodological framework that draws on available research at local scales to arrive at regional and global estimates, while highlighting existing knowledge gaps. The ultimate goal of this study is to bring Indigenous issues to the forefront of ocean sustainability and food sovereignty discussions, providing quantitative data to update our understanding of diversity in global marine management and research, and stimulate further directed research at multiple scales.

## Methods

### Definition of “coastal Indigenous peoples”

A universal definition of “Indigenous peoples” is considered unnecessary and undesirable by both Indigenous peoples and international organizations [34]. Indigenous communities can include government-recognized and unrecognized Indigenous groups, and/or minorities who are indigenous to a region. The most widely used working definition, developed by the United Nations [35], characterizes Indigenous communities, peoples, and nations, as “having a historical continuity with pre-invasion and pre-colonial societies that developed on their territories [and] consider themselves distinct from other sectors[.] They form […] non-dominant sectors […] determined to preserve, develop and transmit to future generations their ancestral territories, and their ethnic identity, as the basis of their continued existence as peoples…”.

**Fig 1:**
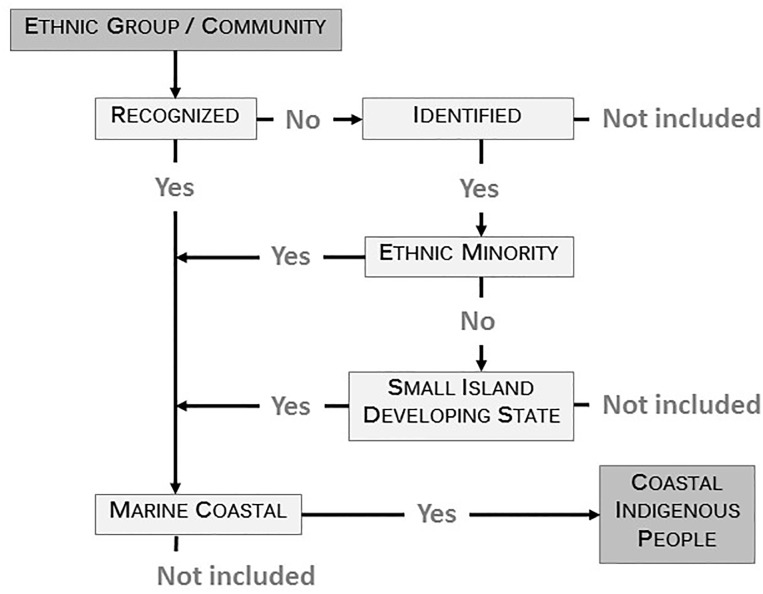
Definition of “coastal Indigenous people” study unit. Only for the purposes of this analysis, a Coastal Indigenous People (CIP) is a recognized or self-identified ethnic minority (or Small Island Developing State) community or group residing on a marine coast.

Following from the above, and solely for the purposes of this study, “coastal Indigenous peoples” (CIPs) here include recognized Indigenous groups, and unrecognized but self-identified ethnic minority groups, whose cultural heritage and socio-economic practices are connected to marine ecosystems that are central to their daily lives and key to their nature-culture dynamics and concepts of surroundings, language, and world views [36,37] (Fig 1).

### Small Island Developing States

Although they are often not demographic minorities, Indigenous peoples in Small Island Developing States (SIDS) are included in the above definition as their relationships to surrounding marine environments hold similar social and cultural importance to CIPs as defined above. With the exception of distinctively highland groups (e.g., in Papua New Guinea), populations in SIDS are thus inextricably linked with marine ecosystems. We therefore include ethnic Indigenous populations (based on census data) of the SIDS as CIPs regardless of whether they are minorities, though we present these results separately to maintain consistency in our method (Fig 1). For instance, in Fiji, we include ethnic Melanesians and Polynesians but not European or Indo-Fijian residents. Where appropriate, as in the case of the Caribbean, we only include recognized extant Indigenous populations (for example, the Carib community in Trinidad and Tobago).

### List of coastal Indigenous peoples

Following from the above (Fig 1), a global list of CIPs was compiled comprising two categorical units. The first represents unique ethnic groups identified as CIPs, whereas the second further specifies community-level (e.g., town or village) information where available. This list was compiled from Yale University’s eHRAF World Cultures database [38], the Minority Rights Group’s database [39], UN Indigenous Peoples Working Group [40], ethnographic and ethnolinguistic maps (e.g., [41]), national census data (e.g., [42–44], governmental and non-governmental organizations [45,46], and complementary systematic Internet searches. Then, we further sought the advice of local researchers to review and confirm the inclusion of corresponding regional CIPs. During the development of our database and subsequent analysis, we aimed to be inclusive of groups and associated information, yet conservative in our quantitative estimates. For each CIP record, key attributes include the group (i.e., a unique group can have multiple communities), population, and location. Attributes reported at the community level (e.g., ethnographic information) were adopted for the corresponding group.

### Estimation of seafood consumption

Information sources on CIPs were examined for data related to seafood (here meant as fish, invertebrates, and other marine living resources) consumption at the group or community scale. This included reviewing available ethnographies and public health data and publications on CIPs to find quantitative information on seafood consumption. Conversely, quantitative research on fisheries catch was examined to identify subsistence catch of CIPs specifically. The data included represent direct measures of consumption (e.g., from diet or subsistence fishery studies) rather than inferences based on production and net trade (i.e., ‘apparent consumption’). All references for data included in the analysis are presented in S1 Text.

For each CIP with seafood consumption data, we calculated the ratio between per capita consumption and the per capita consumption of the country (or, for transboundary groups, the subregion) where it is located. This ratio addresses potential inter-country differences in fish consumption due to wider environmental, cultural, or social characteristics, and is used as a multiplier (*M*) on country-level consumption per capita in subsequent estimates. Note that seafood required for consumption does not account for fish sold, exported, or traded, and therefore represents the minimum seafood required by each CIP.

When community-specific data were not available, a meta-analytical value-transfer model was used to estimate seafood consumption. This is a step-wise approach to estimating missing data, under the key assumption that missing data points can be imputed using available data from similar cases [47]. The main criticism of meta-analyses is that this key assumption is not always met [48–50], and that the smaller-scale research studies on which the analysis depends can themselves be biased [48–51]. Nevertheless, meta-analyses remain crucial for addressing urgent large-scale issues when standardized data collection is absent [48,52], and have been applied, for example, in global assessments of ecosystem services [53], marine ecotourism [54], and factors contributing to various human health risks [55,56]. A transparent discussion of model assumptions allows for subsequent improvements as new data or insights emerge, and fostering such discussion and research is indeed one of our main objectives. Sensitivity analyses are presented in S2 Text.

Grouping records into meaningful socio-economic, political, geographic, and ecological sets is key for this analysis, so we used UN-defined ocean areas and global regions [57,58]. Thus, when specific data were not available, *M* was used as the mean of available data in the corresponding *i*) ethnic group; *ii*) country; *iv*) subregion; *v*) ocean area (FAO Major Fishing Area); or *vi*) region, in that order. Total seafood consumption (S) for all CIPs (*i*) was calculated as:

$$S=\sum_{i}\left(P_{i}\;\cdot \;CC_{i}\;\cdot \;M_{i}\right)$$ (1)

where *P* is the population, *CC* is the corresponding country consumption, and *M* is the corresponding consumption ratio. For large countries spanning more than one ocean area (e.g., Australia, Canada, Mexico, Russia, USA), these areas come before country-level aggregation of *M*; e.g., groups in the Canadian Arctic share more similar environment and customs among each other than to those of the Canadian Pacific or Atlantic coasts. To test the assumption that there are similarities between fish consumption of different groups in similar ecological and geographic settings, we perform a Tukey HSD (Honest Significant Difference) test for significant differences between global subregions with available consumption data.

Population data were often available, but, where absent, we assumed that the population was equal to half of the value of the first quantile for CIPs with available population data. This assumes that communities or groups without any available population estimates are most likely small. This very conservative estimate may be a source of underestimation of results, yet is necessary given the scope of the analysis and can easily be updated as data become available. To provide a range for global estimates, the standard deviation of estimated yearly fish consumption is used for CIPs where data are available, and the mean of all standard deviation data is used for CIPs without such data.

In order to test the sensitivity of consumption estimates to starting data, we use a jackknife approach [59] and run the meta-analytical model 5,000 times omitting a random subset of 10% of observed (i.e., reference) data points on each iteration. We also tested the sensitivity of the above jackknifing method to the total number of initial data points excluded from the model on each iteration (i.e., the 10% value used above). The resulting coefficient of variation (CV) in consumption estimates was then calculated by subregion, region, and at the global level. Finally, we tested the accuracy of individual data point estimates by excluding one data point at a time from the model and comparing the estimated consumption per capita for that data point to the known value. Key results are discussed below, with further supporting figures and tables in S2 Text.

## Results

**Fig 2:**
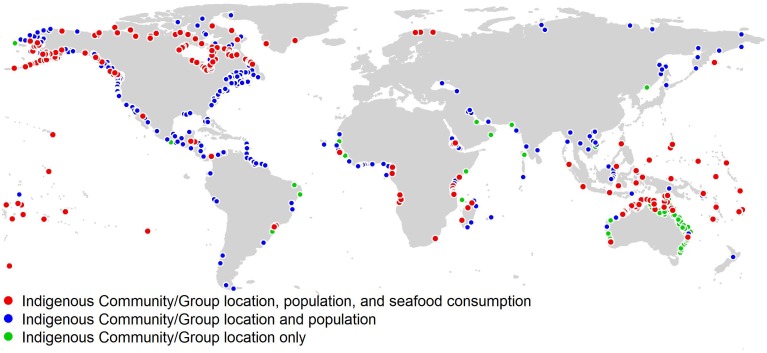
Locations of coastal Indigenous peoples (community or group-level) included in the database. Colors denote the amount of data available for each CIP (i.e., location, population, seafood consumption rate).

The coastal Indigenous group database includes 1,924 CIP records and 611 unique groups; 87 countries are represented, spanning all five FAO regions and 20 of the 21 FAO subregions. Fish consumption data were found for 156 CIPs belonging to 110 Indigenous groups, representing over 3.5 million people. CIP locations are shown in Fig 2, including the data found for each, i.e. group location, population, and fish consumption rate (*n* = 308), group location and population (*n* = 1,198), and group location only (*n* = 418). The average year for reported data was 2003 (min. 1962, max. 2015; σ = 10.5), with 38% corresponding to 2010 or later.

Seafood consumption data at the community or group level were available for 16% of CIPs (including SIDS). For the meta-analytical estimation, 73% of CIPs had data available at the country level (or, for large countries, ocean area) and 10% at the sub-region level; only 1% of CIP consumption estimates for smaller countries were based on data at the ocean area level; use of regional-level estimates was not necessary.

**Fig 3:**
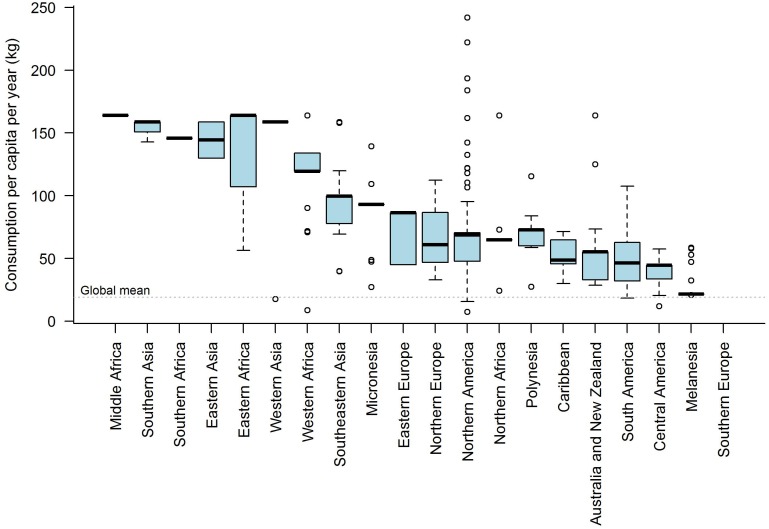
Consumption per capita for Indigenous groups by global subregion. Values are average over all CIPs in each subregion, including both primary and estimated data. Global mean consumption per capita (dashed line) is for world population based on FAO data [60].

There were significant differences in consumption per capita ratios (Tukey HSD, α = 0.05; S1 Table) across global subregions (Fig 3), supporting our meta-analysis grouping categories. The median population of CIPs with available data is 1,308; half of the first quantile is 200, the value used for CIPs without population data.

**Fig 4:**
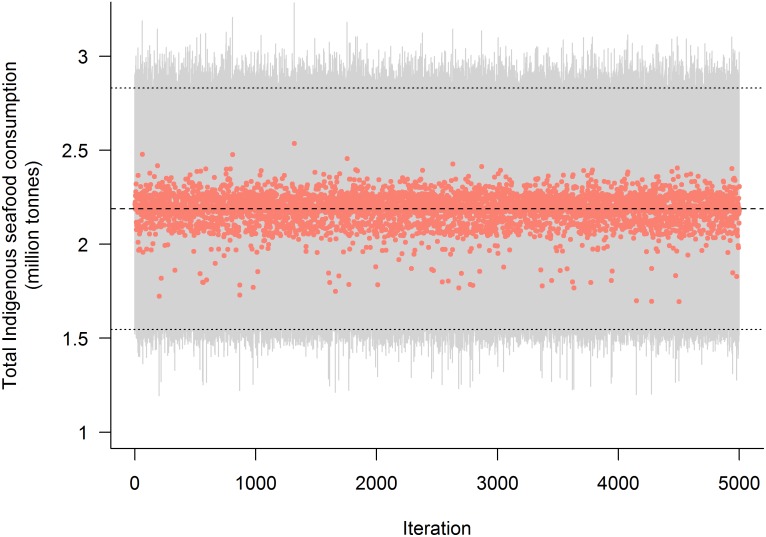
Estimated total global Indigenous food consumption given initial data used. For each of 5,000 model runs (points), each model iteration randomly omits 10% of initial (observed) data points. Grey area is the confidence bound for each model estimation. Dashed lines show mean (2.1 million tonnes), lower (1.5 million tonnes), and upper (2.8 million tonnes) consumption estimates averaged over all iterations.

Based on available data, we estimate that 26.6 million coastal Indigenous peoples globally account for a yearly seafood consumption of 1.9 million (1.3 million—2.5 million). Indigenous coastal inhabitants of SIDS consume a further 231 thousand tonnes (167 thousand—295 thousand) of seafood per year. Result from sensitivity analyses show that the range of consumption estimates given fewer initial data was narrow (CV = 3.6%), with a tendency to underestimate consumption relative to the baseline estimate using all available data (Fig 4). As expected, regions with the least number of observed data (Table 1) had higher uncertainty in consumption estimates (CV = 4–14%; S2 Text, S2 Table).

**Table 1:** Indigenous fish consumption rate, consumption rate relative to country, and total yearly consumption. Values are mean, or total, for all CIPs (*N*) by region; *n* is the number of data points by region. For transboundary groups, consumption ratios are relative to subregion country average.

Region	n	N	Fish consumption (kg·capita^-1^·year^-1^)	Consumption ratio	Total consumption (t 10^3^·year^-1^)
*Africa*	*24*	*133*	*109*	*63*	*826 (±247)*
Eastern Africa	11	28	142	25.9	315 *(±96)*
Middle Africa	5	18	164	9.7	50 *(±15)*
Northern Africa	6	44	67	162	26 *(±8)*
Southern Africa	1	1	145	25.5	0.03 *(±0*.*01)*
Western Africa	1	42	107	7.4	436 *(±128)*
*Americas*	*119*	*443*	*65*	*3*.*2*	*174 (±50)*
Caribbean	1	5	52	2.4	1 *(±0*.*4)*
Central America	3	28	39	3.8	76 *(±22)*
Northern America	111	374	69	3.1	41 *(±11)*
South America	4	36	47	3.5	56 *(±16)*
*Asia*	*9*	*184*	*90*	*7*.*9*	*955 (±285)*
Eastern Asia	-	2	144	3.8	7 *(±2)*
Southeastern Asia	9	167	91	2.6	164 *(±53)*
Southern Asia	-	8	90	71.3	777 *(±229)*
Western Asia	-	7	80	61.6	6 *(±2)*
*Europe*	*4*	*22*	*70*	*3*.*0*	*24 (±7)*
Eastern Europe	1	19	71	3.2	14 *(±4)*
Northern Europe	3	3	69	1.6	10 *(±3)*
*Oceania*	*152*	*1,142*	*33*	*1*.*6*	*213 (±58)*
Australia & New Zealand	119	332	48	1.8	47 *(±14)*
Melanesia	15	758	24	1.6	100 *(±28)*
Micronesia	9	29	85	1.5	30 *(±8)*
Polynesia	9	23	66	1.1	35 *(±8)*
**Global**	**308**	**1,924**	**74**	**15.7**	**2,192** *(±647)*

Decreasing the total number of data points included in the analysis was directly correlated with increases in the CV of total consumption estimates, but also with relatively small decreases in estimated total consumption (S2 Text, S1 Fig). This reflects our systematic conservative assumptions throughout the estimation method, and is also evident when comparing estimates of specific data points with known data, where the highest known consumption values are consistently underestimated (S2 Fig).

The average annual fish consumption per capita (in kg, henceforth ‘kyc’) for groups in the database was 74 kyc (Table 1), compared with a global average (national-level) consumption of 19 kyc estimated by the FAO [60,61]. Consumption was invariably higher for CIPs compared with their country, or region for transboundary CIPs (Table 1). By region, Africa had the highest mean consumption (109 kyc), and Oceania the lowest (33 kyc) (mainly due to effect of low fish consumption in Papua New Guinea).

**Fig 5:**
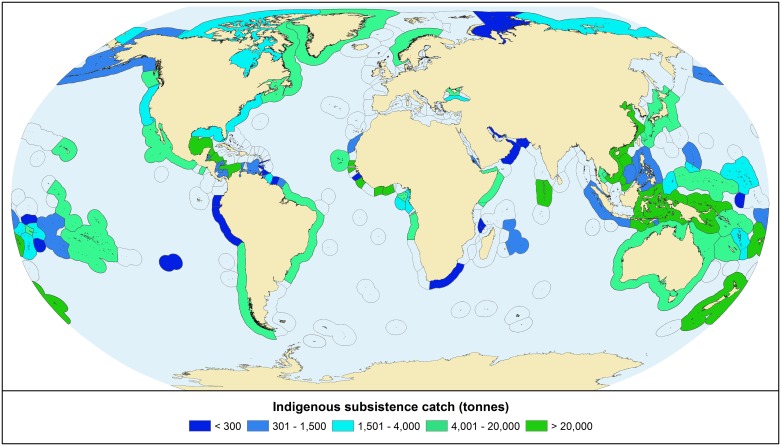
Seafood consumption (tonnes per year) by coastal Indigenous peoples. Values for individual CIPs are summed by Exclusive Economic Zones.

**Fig 6:**
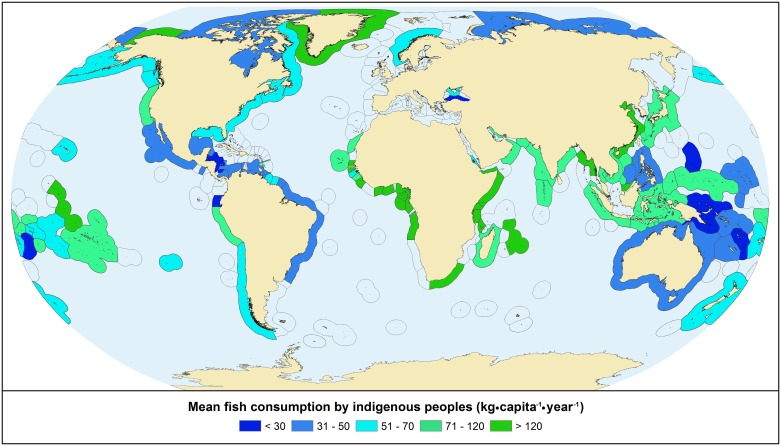
Marine fish consumption per capita (kg·year^-1^) by coastal Indigenous peoples. Values for individual CIPs are averaged by Exclusive Economic Zone.

The highest total consumption occurs in tropical areas with large coastal Indigenous populations and high per capita consumption rates (Fig 5). Nevertheless, Arctic CIPs have the highest mean per capita consumption by climate region, at 74 kyc, followed by the CIPs of temperate (66 kyc) and tropical (47 kyc) regions (Fig 6).

## Discussion

The scale of coastal Indigenous seafood consumption is highly relevant to international policies for the recognition and protection of Indigenous peoples’ livelihoods, and significant for fisheries management at international and national scales. Drawing from research in various disciplines, a wealth of information exists to inform this and future interdisciplinary studies on Indigenous issues, highlighted by the wide range of case studies found across the world (Fig 2; S1 Text), representing over 3.5 million Indigenous peoples. Based on this data, Indigenous seafood consumption is estimated at approximately 1.9 million (1.3 million—2.5 million) tonnes per year, plus 231 thousand tonnes (167 thousand—295 thousand) tonnes by Indigenous populations of Small Island Developing States. Total estimated seafood requirements by Indigenous peoples are thus equal to 1–2% of global catch [61,62]. It must also be stressed that, in addition to providing conservative estimates of seafood consumption, due to data availability we did not include commercial fishery catch that is often vital for Indigenous economies [63,64]. Therefore, our results must be viewed as a bare minimum estimate of Indigenous fish requirements that does not reflect employment and economic needs.

Seafood consumption rates among coastal Indigenous peoples (CIPs) (74 kg·capita^-1^·year^-1^) are, unsurprisingly, higher than national averages (19 kg·capita^-1^·year^-1^), though comparisons between consumption of Indigenous peoples and the regions in which they are located (Table 1) reflect the crucial relative importance of seafood for these communities. There is also some evidence of higher proportion of personal consumption of catch by Indigenous compared to non-Indigenous commercial fishers operating in the same area (based on data in [65]). However, such comparisons are beside the primary goal of recognizing the critical role of marine living resources in supporting Indigenous food sovereignty and cultural use, consistent with international agreements including the UN Declaration on the Rights of Indigenous Peoples calling for “sustainable and equitable development and proper management of the environment” [23] and the UN Sustainable Development Goals designed to promote ecological and economic sustainability while acknowledging and protecting the needs of vulnerable human populations [66].

Total and per-capita seafood demand of CIPs is concentrated around equatorial regions in Africa and Asia, and in the Arctic (Figs 5 and 6) where marine living resources are subject to mounting pressure from foreign and domestic fishing fleets [67,68], new transport routes [69], marine non-living resource claims, and climate change [8,70,71]. Furthermore, many Indigenous communities are in regions without strong governance or protections against such pressures (Fig 2; [72]). Catch by Indigenous fisheries should be taken into account by resource management policies, yet we argue that although their total catch is significant for communities, curtailing large-scale overexploitation of the world’s oceans must first address overcapacity in commercial fishing fleets and other exogenous pressures [73].

As international environmental governance advances (e.g., 21), management decisions affecting Indigenous peoples should require their involvement and consent. The aggregated data presented here are no more important than the understanding developed from direct engagement with Indigenous peoples. It is, rather, a necessary step to add value to ongoing research by consolidating a platform for cross-scale and interdisciplinary discussion. This will increasingly rely both on continued ethnographic and public health studies, and integration of Indigenous issues in quantitative analyses regarding fisheries and coastal management policies [10].

Qualitative research on Indigenous fisheries has provided crucial perspectives by addressing their cultural meaning and social organization. Applied studies on broader small-scale fisheries usually explore the engagement of local fishers with management or conservation strategies (e.g., [74,75]), and may contain fisheries data such as gear type, catch, and fisher populations. Both approaches contribute to general understanding of resource commons and economies centered on fisheries, yet are often focused on local scales [32], can overemphasize particular social-ecological feedbacks [76], and may not be able to accommodate multiple dynamics and impacts of ecological and economic change [32]. Nevertheless, these studies are vital for informing international (through cross-scale analyses) and local policies that are required to improve conditions in coastal communities. A key qualitative observation based on our review of coastal Indigenous fisheries (S1 Text) is that traditional ecological knowledge plays a vital role for both local resource management and social dynamics. However, global ecological and economic changes are rapidly overtaking communities’ ability to resist and adapt to combined pressures, making estimates of scale vital for addressing issues from multiple perspectives.

A key benefit of our methodological framework is that it can be refined through examination of assumptions, added data, and independent estimates. Sensitivity analyses (Fig 4, and S2 Text, S1 and S2 Figs) show that the model is robust to uncertainty regarding initial data, and tends to slightly underestimate consumption when data are not available (S1 Fig). In the case of Australia, Kleisner et al. [77] provide a wholly independent estimate of Indigenous subsistence fisheries catch that, due to issues of aggregation scale, we were unable to directly integrate into our data. The authors of said study estimate Indigenous subsistence catch for Australia at 3,482 t·year^-1^, compared with our meta-analysis-based consumption estimate of 3,180–5,726 t·year^-1^. This is encouraging in terms of validating and refining our model as more data become available. Importantly, our findings highlight regional (e.g., Fig 2, S2 Table) and thematic gaps in available data that could be addressed through subsequent targeted research.

We note that this global overview requires collecting and categorizing group and community-level information without fully reflecting its historical context, which can be controversial when group identity and membership continue to be exogenously and endogenously contested. For example, ethnicity in some regions (e.g., India) is tied to social class struggles and discrimination, an issue which we do not address here. Moreover, applying fisheries and economics concepts to interpret cultural uses of fish can make our estimates less appropriate in terms of defining the practice of fishing from a cultural perspective. If fish are not caught or exchanged in a “traditional manner” or “ceremonial ritual”, for instance, or if “traditional ecological knowledge” is not directly applied, should this fishery be considered less culturally important? These issues are beyond the scope of this study, yet can be addressed through further integration of ethno-historical contexts in research, despite being unlikely to ever address in general terms all complexities of cultural identities [35,78].

International environmental sustainability strategies identify a need to recognize and support Indigenous fisheries and their contribution to culture and food sovereignty [24,25]; the results of this study provide scale to these multiple challenges. Innumerable research questions arise through reviews of Indigenous fisheries including, for example, the interplay between contaminant exposure and nutritional benefits of seafood consumption (e.g., [22,79]); the internal dynamics of larger ethnic groups (including those of the SIDS); the dynamics of commercial (including Indigenous) and non-commercial fisheries; the role of Indigenous traditional ecological knowledge (e.g., [80]); and the impacts of climate change on coastal Indigenous communities through effects on ecosystems and culturally iconic species (e.g., [10]). By explicitly adding quantitative context to Indigenous issues, as done here, these questions can be addressed in an interdisciplinary manner incorporating perspectives from ecology, economics, public health, etc., in addition to qualitative research.

This study provides quantitative scale to marine Indigenous fisheries, while reinforcing the argument that the recognition of community practices—a defining characteristic of Indigenous peoples—is key to sustainable fisheries and oceans. The scale of ongoing ecological and socioeconomic challenges cannot be remediated by the actions of Indigenous peoples alone. Coastal Indigenous communities require increased recognition and support from the public and legitimate organizations, as well as interdisciplinary analyses that carefully consider cultural value and perspective as well as global ecological and economic trends.

Finally, we argue that recognizing the importance of access to natural resources for Indigenous peoples contributes to ensuring their human security. Virtually all groups reviewed in this study contend with resource competition with other communities, international corporations, and states, but also face security risks through broader conflicts and displacement. Threats to their security stem from both historical and contemporary dynamics, and are further exacerbated by risks from climate change [81] as well as limitations in international governance of coastal development and maritime industries [82]. In creating paths for coastal Indigenous peoples to secure their access to seafood, the international community faces the urgent task of protecting not just fish and marine ecosystems but the peoples themselves, that is, the lives and cultures which shape our oceans.

## Supporting Information

S1 Fig Estimated Indigenous seafood consumption given assumptions on initial available data. For each exclusion ratio, a different random subset of initial data points was excluded from the analysis in each of 1,000 model runs. Points show mean consumption estimate; lines show coefficient of variation.
(TIF)

S2 Fig Observed and estimated consumption values for initial data points. Each observed data point was omitted from model input and estimated from remaining data. Solid line is 1:1.
(TIF)

S1 Table Comparison of consumption data across global subregions. Tukey HSD test (α = 0.05) results comparing average seafood consumption ratio per capita by global subregion. Only primary data, not estimates, are used in this analysis. Pairs marked with asterisks have significantly different seafood consumption (*p<0.05; **p<0.01).
(DOCX)

S2 Table Number of data points (*n*), total number of coastal Indigenous peoples (CIPs) (N), data points as percentage of total (n/N), and coefficient of variation (CV). CV is estimated based on 5,000 model runs, where a random 10% of data points are excluded from the analysis before each iteration.
(DOCX)

S1 Text Data references for Indigenous seafood consumption. References can contain information for more than one coastal Indigenous community and/or group.
(DOCX)

S2 Text Sensitivity analysis methods and implications for seafood consumption estimates. (DOCX)
