# Supplementary figures and images for: A Global Estimate of Seafood Consumption by Coastal Indigenous Peoples
Source: PLoS One. 2016 Dec 5;11(12):e0166681. doi: 10.1371/journal.pone.0166681 (PMC5137875; doi:10.1371/journal.pone.0166681)

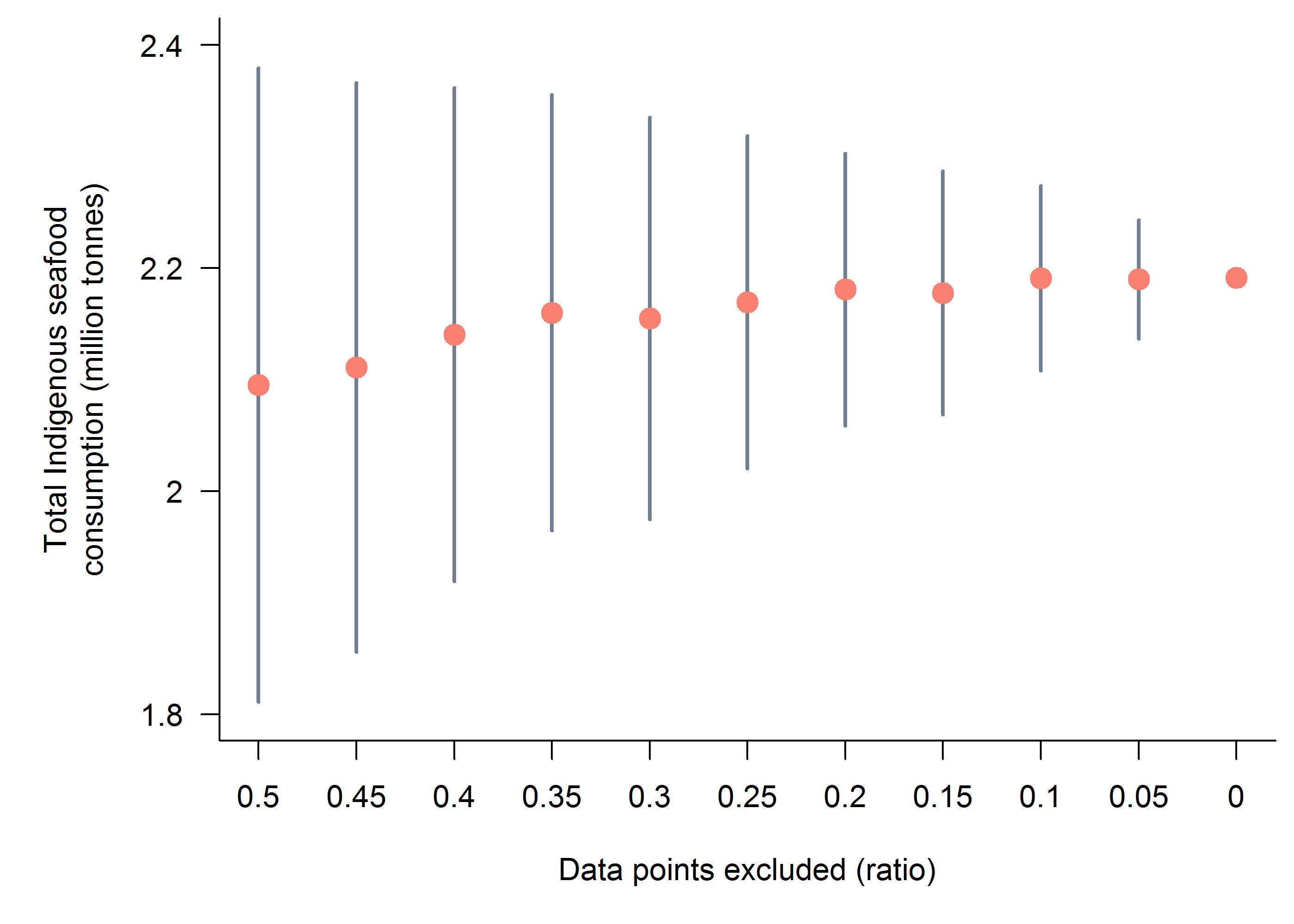

Supplement: S1 Fig — For each exclusion ratio, a different random subset of initial data points was excluded from the analysis in each of 1,000 model runs. Points show mean consumption estimate; lines show coefficient of variation. (TIF) [file pone.0166681.s001.tif]

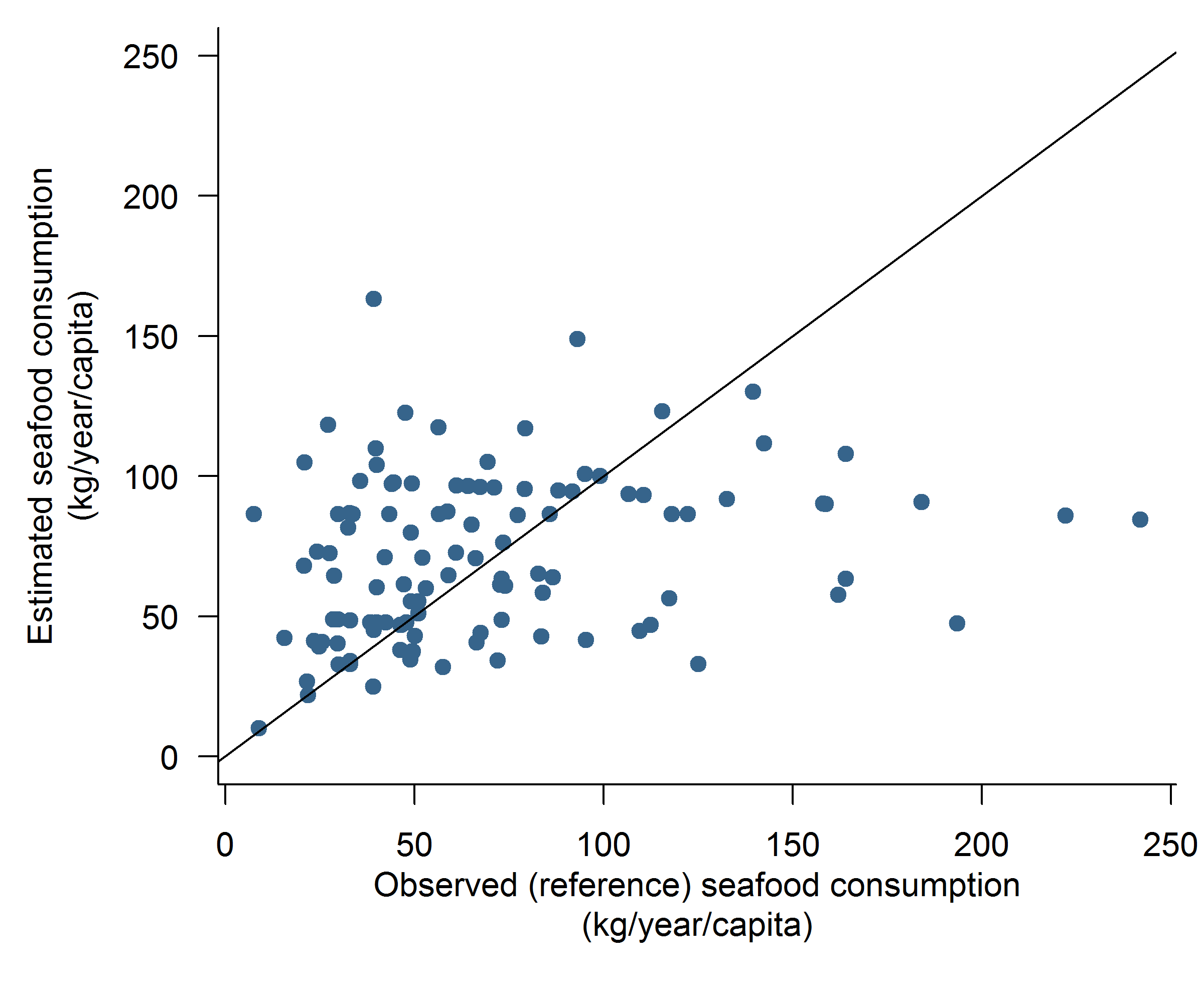

Supplement: S2 Fig — Each observed data point was omitted from model input and estimated from remaining data. Solid line is 1:1. (TIF) [file pone.0166681.s002.tif]
