# Supplementary material for: A Global Estimate of Seafood Consumption by Coastal Indigenous Peoples
Source: PLoS One. 2016 Dec 5;11(12):e0166681. doi: 10.1371/journal.pone.0166681 (PMC5137875; doi:10.1371/journal.pone.0166681)
Supplement: S1 Table — Tukey HSD test (α = 0.05) results comparing average seafood consumption ratio per capita by global subregion. Only primary data, not estimates, are used in this analysis. Pairs marked with asterisks have significantly different seafood consumption (*p<0.05; **p<0.01). (DOCX) [file pone.0166681.s003.docx]

**S3 Table. Comparison of consumption data across global subregions.** Tukey HSD test (α=0.05) results comparing average seafood consumption ratio per capita by global subregion. Only primary data, not estimates, are used in this analysis. Pairs marked with asterisks have significantly different seafood consumption (*p<0.05; **p<0.01).

| **Subregion pair** | **p (adjusted)** | |
| --- | --- | --- |
| Australia and New Zealand-Caribbean | 1.00 |  |
| Australia and New Zealand-Eastern Africa | 0.00 | ** |
| Australia and New Zealand-Eastern Asia | 0.00 | ** |
| Australia and New Zealand-Eastern Europe | 0.00 | ** |
| Australia and New Zealand-Micronesia | 0.00 | ** |
| Australia and New Zealand-Middle Africa | 0.00 | ** |
| Australia and New Zealand-Northern Africa | 0.00 | ** |
| Australia and New Zealand-Northern America | 0.00 | ** |
| Australia and New Zealand-Northern Europe | 0.95 |  |
| Australia and New Zealand-Polynesia | 0.00 | ** |
| Australia and New Zealand-Southeastern Asia | 0.00 | ** |
| Australia and New Zealand-Southern Africa | 0.00 | ** |
| Australia and New Zealand-Southern Asia | 0.00 | ** |
| Australia and New Zealand-Western Africa | 0.00 | ** |
| Australia and New Zealand-Western Asia | 0.00 | ** |
| Caribbean-Eastern Africa | 0.00 | ** |
| Caribbean-Eastern Asia | 0.00 | ** |
| Caribbean-Eastern Europe | 0.94 |  |
| Caribbean-Micronesia | 0.08 |  |
| Caribbean-Middle Africa | 0.00 | ** |
| Caribbean-Northern Africa | 0.99 |  |
| Caribbean-Northern America | 0.95 |  |
| Caribbean-Northern Europe | 1.00 |  |
| Caribbean-Polynesia | 1.00 |  |
| Caribbean-Southeastern Asia | 0.00 | ** |
| Caribbean-Southern Africa | 0.00 | ** |
| Caribbean-Southern Asia | 0.00 | ** |
| Caribbean-Western Africa | 0.00 | ** |
| Caribbean-Western Asia | 0.00 | ** |
| Central America-Australia and New Zealand | 0.80 |  |
| Central America-Caribbean | 1.00 |  |
| Central America-Eastern Africa | 0.00 | ** |
| Central America-Eastern Asia | 0.00 | ** |
| Central America-Eastern Europe | 0.00 | ** |
| Central America-Micronesia | 0.00 | ** |
| Central America-Middle Africa | 0.00 | ** |
| Central America-Northern Africa | 0.00 | ** |
| Central America-Northern America | 0.00 | ** |
| Central America-Northern Europe | 0.62 |  |
| Central America-Polynesia | 0.00 | ** |
| Central America-South America | 0.99 |  |
| Central America-Southeastern Asia | 0.00 | ** |
| Central America-Southern Africa | 0.00 | ** |
| Central America-Southern Asia | 0.00 | ** |
| Central America-Western Africa | 0.00 | ** |
| Central America-Western Asia | 0.00 | ** |
| Eastern Africa-Eastern Asia | 1.00 |  |
| Eastern Africa-Middle Africa | 0.04 | * |
| Eastern Africa-Southern Africa | 1.00 |  |
| Eastern Africa-Southern Asia | 0.99 |  |
| Eastern Asia-Middle Africa | 1.00 |  |
| Eastern Asia-Southern Africa | 1.00 |  |
| Eastern Asia-Southern Asia | 1.00 |  |
| Eastern Europe-Eastern Africa | 0.00 | ** |
| Eastern Europe-Eastern Asia | 0.00 | ** |
| Eastern Europe-Micronesia | 0.67 |  |
| Eastern Europe-Middle Africa | 0.00 | ** |
| Eastern Europe-Southeastern Asia | 0.01 | * |
| Eastern Europe-Southern Africa | 0.03 | * |
| Eastern Europe-Southern Asia | 0.00 | ** |
| Eastern Europe-Western Africa | 0.00 | ** |
| Eastern Europe-Western Asia | 0.00 | ** |
| Melanesia-Australia and New Zealand | 0.00 | ** |
| Melanesia-Caribbean | 0.17 |  |
| Melanesia-Central America | 0.01 | * |
| Melanesia-Eastern Africa | 0.00 | ** |
| Melanesia-Eastern Asia | 0.00 | ** |
| Melanesia-Eastern Europe | 0.00 | ** |
| Melanesia-Micronesia | 0.00 | ** |
| Melanesia-Middle Africa | 0.00 | ** |
| Melanesia-Northern Africa | 0.00 | ** |
| Melanesia-Northern America | 0.00 | ** |
| Melanesia-Northern Europe | 0.02 | * |
| Melanesia-Polynesia | 0.00 | ** |
| Melanesia-South America | 0.00 | ** |
| Melanesia-Southeastern Asia | 0.00 | ** |
| Melanesia-Southern Africa | 0.00 | ** |
| Melanesia-Southern Asia | 0.00 | ** |
| Melanesia-Western Africa | 0.00 | ** |
| Melanesia-Western Asia | 0.00 | ** |
| Micronesia-Eastern Africa | 0.00 | ** |
| Micronesia-Eastern Asia | 0.01 | * |
| Micronesia-Middle Africa | 0.00 | ** |
| Micronesia-Southeastern Asia | 1.00 |  |
| Micronesia-Southern Africa | 0.22 |  |
| Micronesia-Southern Asia | 0.00 | ** |
| Micronesia-Western Africa | 0.00 | ** |
| Micronesia-Western Asia | 0.00 | ** |
| Northern Africa-Eastern Africa | 0.00 | ** |
| Northern Africa-Eastern Asia | 0.00 | ** |
| Northern Africa-Eastern Europe | 1.00 |  |
| Northern Africa-Micronesia | 0.03 | * |
| Northern Africa-Middle Africa | 0.00 | ** |
| Northern Africa-Northern America | 1.00 |  |
| Northern Africa-Northern Europe | 1.00 |  |
| Northern Africa-Southeastern Asia | 0.00 | ** |
| Northern Africa-Southern Africa | 0.01 | * |
| Northern Africa-Southern Asia | 0.00 | ** |
| Northern Africa-Western Africa | 0.00 | ** |
| Northern Africa-Western Asia | 0.00 | ** |
| Northern America-Eastern Africa | 0.00 | ** |
| Northern America-Eastern Asia | 0.00 | ** |
| Northern America-Eastern Europe | 1.00 |  |
| Northern America-Micronesia | 0.00 | ** |
| Northern America-Middle Africa | 0.00 | ** |
| Northern America-Northern Europe | 1.00 |  |
| Northern America-Southeastern Asia | 0.00 | ** |
| Northern America-Southern Africa | 0.02 | * |
| Northern America-Southern Asia | 0.00 | ** |
| Northern America-Western Africa | 0.00 | ** |
| Northern America-Western Asia | 0.00 | ** |
| Northern Europe-Eastern Africa | 0.00 | ** |
| Northern Europe-Eastern Asia | 0.01 | * |
| Northern Europe-Eastern Europe | 1.00 |  |
| Northern Europe-Micronesia | 1.00 |  |
| Northern Europe-Middle Africa | 0.00 | ** |
| Northern Europe-Southeastern Asia | 0.93 |  |
| Northern Europe-Southern Africa | 0.09 |  |
| Northern Europe-Southern Asia | 0.00 | ** |
| Northern Europe-Western Africa | 0.11 |  |
| Northern Europe-Western Asia | 0.00 | ** |
| Polynesia-Eastern Africa | 0.00 | ** |
| Polynesia-Eastern Asia | 0.00 | ** |
| Polynesia-Eastern Europe | 1.00 |  |
| Polynesia-Micronesia | 0.09 |  |
| Polynesia-Middle Africa | 0.00 | ** |
| Polynesia-Northern Africa | 1.00 |  |
| Polynesia-Northern America | 1.00 |  |
| Polynesia-Northern Europe | 1.00 |  |
| Polynesia-Southeastern Asia | 0.00 | ** |
| Polynesia-Southern Africa | 0.01 | * |
| Polynesia-Southern Asia | 0.00 | ** |
| Polynesia-Western Africa | 0.00 | ** |
| Polynesia-Western Asia | 0.00 | ** |
| South America-Australia and New Zealand | 1.00 |  |
| South America-Caribbean | 1.00 |  |
| South America-Eastern Africa | 0.00 | ** |
| South America-Eastern Asia | 0.00 | ** |
| South America-Eastern Europe | 0.00 | ** |
| South America-Micronesia | 0.00 | ** |
| South America-Middle Africa | 0.00 | ** |
| South America-Northern Africa | 0.00 | ** |
| South America-Northern America | 0.00 | ** |
| South America-Northern Europe | 0.96 |  |
| South America-Polynesia | 0.05 |  |
| South America-Southeastern Asia | 0.00 | ** |
| South America-Southern Africa | 0.00 | ** |
| South America-Southern Asia | 0.00 | ** |
| South America-Western Africa | 0.00 | ** |
| South America-Western Asia | 0.00 | ** |
| Southeastern Asia-Eastern Africa | 0.00 | ** |
| Southeastern Asia-Eastern Asia | 0.02 | * |
| Southeastern Asia-Middle Africa | 0.00 | ** |
| Southeastern Asia-Southern Africa | 0.37 |  |
| Southeastern Asia-Southern Asia | 0.00 | ** |
| Southeastern Asia-Western Africa | 0.00 | ** |
| Southeastern Asia-Western Asia | 0.00 | ** |
| Southern Africa-Middle Africa | 1.00 |  |
| Southern Africa-Southern Asia | 1.00 |  |
| Southern Asia-Middle Africa | 1.00 |  |
| Western Africa-Eastern Africa | 0.00 | ** |
| Western Africa-Eastern Asia | 0.55 |  |
| Western Africa-Middle Africa | 0.00 | ** |
| Western Africa-Southern Africa | 0.94 |  |
| Western Africa-Southern Asia | 0.00 | ** |
| Western Africa-Western Asia | 0.02 | * |
| Western Asia-Eastern Africa | 1.00 |  |
| Western Asia-Eastern Asia | 1.00 |  |
| Western Asia-Middle Africa | 0.30 |  |
| Western Asia-Southern Africa | 1.00 |  |
| Western Asia-Southern Asia | 0.99 |  |
