# Supplementary material for: A Global Estimate of Seafood Consumption by Coastal Indigenous Peoples
Source: PLoS One. 2016 Dec 5;11(12):e0166681. doi: 10.1371/journal.pone.0166681 (PMC5137875; doi:10.1371/journal.pone.0166681)
Supplement: S2 Table — CV is estimated based on 5,000 model runs, where a random 10% of data points are excluded from the analysis before each iteration. (DOCX) [file pone.0166681.s004.docx]

**S6 Table. Number of data points (*n*), total number of coastal Indigenous peoples (CIPs) (N), data points as percentage of total (n/N), and coefficient of variation (CV).** CV is estimated based on 5,000 model runs, where a random 10% of data points are excluded from the analysis before each iteration.

| **Region** | **n** | **N** | **n/N (%)** | **CV (%)** |
| --- | --- | --- | --- | --- |
| *Africa* | *24* | *133* | *18* | *7.5* |
| Eastern Africa | 11 | 28 | 39 | 10 |
| Middle Africa | 5 | 18 | 27 | 11 |
| Northern Africa | 6 | 44 | 13 | 10 |
| Southern Africa | 1 | 1 | 100 | 13 |
| Western Africa | 1 | 42 | 2 | 6 |
| *Americas* | *119* | *443* | *27* | *3.7* |
| Caribbean | 1 | 5 | 20 | 6 |
| Central America | 3 | 28 | 11 | 5 |
| Northern America | 111 | 374 | 30 | 6 |
| South America | 4 | 36 | 11 | 8 |
| *Asia* | *9* | *184* | *5* | *5.9* |
| Eastern Asia | - | 2 | 0 | 4 |
| Southeastern Asia | 9 | 167 | 5 | 5 |
| Southern Asia | - | 8 | 0 | 6 |
| Western Asia | - | 7 | 0 | 7 |
| *Europe* | *4* | *22* | *18* | *9.7* |
| Eastern Europe | 1 | 19 | 5 | 14 |
| Northern Europe | 3 | 3 | 100 | 11 |
| *Oceania* | *152* | *1,142* | *13* | *5.1* |
| Australia & New Zealand | 119 | 332 | 36 | 6 |
| Melanesia | 15 | 758 | 2 | 9 |
| Micronesia | 9 | 29 | 31 | 10 |
| Polynesia | 9 | 23 | 39 | 8 |
| **Global** | **308** | **1,924** | **16** | **3.6** |
