# Supplementary material for: A Global Estimate of Seafood Consumption by Coastal Indigenous Peoples
Source: PLoS One. 2016 Dec 5;11(12):e0166681. doi: 10.1371/journal.pone.0166681 (PMC5137875; doi:10.1371/journal.pone.0166681)
Supplement: S1 Text — References can contain information for more than one coastal Indigenous community and/or group. (DOCX) [file pone.0166681.s005.docx]

**S1 Text. Data references for Indigenous seafood consumption.** References can contain information for more than one coastal Indigenous community and/or group.

Alaska Department of Fish and Game. “Harvest Information for Community.” *ADFG Subsistence Hunting and Fishing. Harvest Data and Reports*, 2016. https://www.adfg.alaska.gov/sb/CSIS/index.cfm?ADFG=harvInfo.harvestCommSelComm.

Allen, S., and P. Bartram. “Guam as a Fishing Community.” Honolulu: Pacific Islands Fisheries Science Center, NOAA, 2008.

Bang, H.O., J. Dyerberg, and N. Hjorne. “The Composition of Food Consumed by Greenland Eskimos.” *Acta Medica Scandinavica*, no. 200 (1976): 69–73.

Barnes-Mauthe, M., K.L.L. Oleson, and B. Zafindrasilivonona. “The Total Economic Value of Small-Scale Fisheries with a Characterization of Post-Landing Trends: An Application in Madagascar with Global Relevance.” *Fisheries Research* 147 (October 2013): 175–85.

Comisión Nacional para el Desarrollo de los Pueblos Indígenas. “Consulta a Los Pueblos Indígenas de La Zona Costera Del Golfo de California Referente Al Ordenamiento Ecológico Marino.” México, D.F.: Comisión Nacional para el Desarrollo de los Pueblos Indígenas, 2009.

Cross, H.C. “The Importance of Small-Scale Fishing to Rural Coastal Livelihoods: A Comparative Case-Study in the Bijagós Archipelago Guinea Bissau.” UCL (University College London), 2014.

Delgado-Ramirez, C.E. “Indigenous Food Consumption in Northwestern Mexico.” Personal communication. México: Instituto Nacional de Antropología e Historia, 2015.

———. “Los Pescadores Seri, Yaqui Y Kineños: Un Estudio Comparativo Sobre La Inserción Del Capitalismo En Tres Comunidades Pesqueras Del Golfo de California.” Escuela Nacional de Antropología e Historia, 2009.

Dewailly, E., C. Blanchet, S. Gingras, S. Lemieux, and B.J. Holub. “Fish Consumption and Blood Lipids in Three Ethinic Groups of Québec (canada).” *Lipids* 38, no. 4 (2003): 359–65.

Divovich, E., D. Belhabib, D. Zeller, and D. Pauly. “Eastern Canada, ‘a Fishery with No Clean Hands’: Marine Fisheries Catch Reconstruction from 1950 to 2010.” Working Paper Series. Vancouver: Fisheries Centre, The University of British Columbia, 2015.

Doherty, B., D. Gibson, A. McCrea-Strub, K. Zylich, D. Zeller, and D. Pauly. “Reconstruction of Marine Fisheries Catches for Subarctic Alaska, 1950-2010.” Fisheries Centre Working Paper Series. The University of British Columbia, 2015.

Domingues do Amaral, B. “Fisheries and Fishing Effort at the Indigenous Reserves Ashaninka/Kaxinawá, River Breu, Brazil/Peru.” *Acta Amazonica* 35, no. 2 (2005): 133–44.

Dyspriani, P. “Indigenous Seafood Consumption in Indonesia.” Personal communication. Indonesia: Ministry of Marine Affairs and Fisheries, 2015.

Fundacao Nacional do Indio. “Pescadores Pataxó E Tupinambá Participam de Oficina Da Funai Sobre Cadeia de Valor Do Pescado.” Comunicacao. Brazil: Ministerio da Justica, 2013. http://www.funai.gov.br/index.php/comunicacao/noticias/491-pescadores-pataxo-e-tupinamba-participam-de-oficina-da-funai-sobre-cadeia-de-valor-do-pescado.

Gillete, R. “Fisheries in the Economies of the Pacific Island Countries and Territories.” Pacific Studies Series. Philippines: Asian Development Bank, 2009.

Hanazaki, N., and A. Begossi. “Does Fish Still Matter? Changes in the Diet of Two Brazilian Fishing Communities.” *Ecology of Food and Nutrition* 42, no. 4–5 (June 2003): 279–301.

Harper, S., H.M. Guzman, K. Zylich, and D. Zeller. “Reconstructing Panama’s Total Fisheries Catches from 1950 to 2010: Highlighting Data Deficiencies and Management Needs.” *Marine Fisheries Review* 76, no. 1–2 (June 17, 2014): 51–65.

Harris, A., G. Dews, I. Poiner, and J. Kerr. “The Traditional and Island Based Catch of the Torres Strait Protected Zone. Final Report on CSIRO Research, 1990-1993.” Australia: CSIRO Division of Fisheries, 1994.

Henry, G.W., J.M. Lyle, NSW Fisheries, Australia, and Fisheries and Forestry Department of Agriculture. *The National Recreational and Indigenous Fishing Survey*. Canberra: Australian Government Department of Agriculture, Fisheries and Forestry, 2003.

Hoehn, S., and B. Thapa. “Attitudes and Perceptions of Indigenous Fishermen towards Marine Resource Management in Kuna Yala, Panama.” *International Journal of Sustainable Development & World Ecology* 16, no. 6 (December 2009): 427–37.

Hospital, J., and C. Beavers. “Economic and Social Characteristics of Guam’s Small Boat Fisheries.” Honolulu: Pacific Islands Fisheries Science Center, NOAA, 2012.

Jokelainen, A., M. Pekkarinen, P. Roine, and J.K. Miettinen. “The Diet of Finnish Lapps.” *Zeitschrift Für Ernährungswissenschaft* 3, no. 1–2 (1962): 110–17.

Kilarski, Stacey. “Decision Support for Coral Reef Fisheries Management: Community Input as a Means of Informing Policy in American Samoa.” University of California Santa Barbara, 2006.

Kleisner, K., C. Brennan, A. Garland, S. Lingard, S. Tracey, P. Sahlqvist, A. Tsolos, D. Pauly, and D. Zeller. “Australia: Reconstructing Estimates of Total Fisheries Removals 1950-2012.” Working Paper Series. Fisheries Centre: The University of British Columbia, 2015.

Komilus, C.F., H.C. Ha, and S.I. Nguang. “Component 4: Demonstration of Best Fisheries Management Practices in Critical Sites of the Sulu-Celebes Sea  Activity 3: Gathering Socio-Economic Baseline Indicators at Demonstration Site in Semporna, Malaysia.” Sulu-Celebes Sea Sustainable Fisheries Management Project. Malaysia: Universiti Sultan Zainal Abidin, 2013. http://erep.unisza.edu.my/1965/.

Laird, B. D., A. B. Goncharov, G.M. Egeland, and H.M. Chan. “Dietary Advice on Inuit Traditional Food Use Needs to Balance Benefits and Risks of Mercury, Selenium, and n3 Fatty Acids.” *Journal of Nutrition* 143, no. 6 (June 1, 2013): 923–30. doi:10.3945/jn.112.173351.

MacCord, P.L., A. Begossi. “Dietary Changes over Time in a Caiçara Community from the Brazilian Atlantic Forest.” *Ecology and Society* 11, no. 2 (2006): 38.

Molina-Miranda, J.M.F. “Caracterización Del Patrón Alimentario de La Población Garífuna Que Reside En El Municipio de Livingston, Izabal. Guatemala 2012.” Universidad Rafael Landívar, 2012.

Nietschmann, B. “Indigenous Island Peoples, Living Resources and Protected Areas,” 1–31. Bali, 1982.

Pauletto, P., M. Puato, M.G. Caroli, E.Casiglia, A.E. Munhambo, G. Cazzolato, G. B. Bon, M.T. Angeli, C. Galli, and A.C. Pessina. “Blood Pressure and Atherogenic Lipoprotein Profiles of Fish-Diet and Vegetarian Villagers in Tanzania: The Lugalawa Study.” *The Lancet* 348, no. 9030 (1996): 784–88.

Pomeroy, R.S., M.D. Pido, J.F.A. Pontillas, B.S. Francisco, A.T. White, and G.T. Silvestre. “Evaluation of Policy Options for the Live Reef Food Fish Trade: Focus on the Calamianes Islands and Palawan Province, Philippines, with Implications for National Policy.” Fisheries Improved for Sustainable Harvest Project. Philippines: Palawan Council for Sustainable Development, Provincial Government of Palawan, 2005.

Quatisnuxw First Nation, Weiwaikum First Nation, Ahousaht First Nation, Snuneymuxw First Nation, and Pacheedaht First Nation. “Traditional Seafoods of Vancouver Island First Nations. Balancing Health Benefits with Pollution Risks.” In *Final Report. Proceedings of a Workshop at Malaspina Campus, Vancouver Island University and the Celebration Feast at Snuneymuxw First Nation*, 16. Vancouver Island: Chemainus First Nation, 2008.

Rakotondrazafy, V. “Indigenous Seafood Consumption in Madagascar.” Personal communication. Madagascar: Madagascar Locally Managed Marine Area Network, 2015.

Ramires, M., and W. Barrella. “Ecologia Da Pesca Artesanal Em Populações Caiçaras Da Estação Ecológica de Juréia-Itatins, São Paulo, Brasil.” *Interciencia* 28, no. 4 (2003): 208–13.

Roe, A. “Fishing for Identity: Mercury Contamination and Fish Consumption Among Indigenous Groups in the United States.” *Bulletin of Science, Technology and Society* 23, no. 5 (October 1, 2003): 368–75.

Russell, S., C.A. Sullivan, and A.J. Reichelt-Brushett. “Aboriginal Consumption of Estuarine Food Resources and Potential Implications for Health through Trace Metal Exposure; A Study in Gumbaynggirr Country, Australia.” Edited by A. Chiaradia. *PLOS ONE* 10, no. 6 (June 22, 2015): e0130689. doi:10.1371/journal.pone.0130689.

Salih, S.A. “Report on a Field Trip to Arafale.” Eritrea: Resources and Environment Division, Ministry of Marine Resources, 1995.

———. “Socio-Economic Study on a Dehil Island Community.” Eritrea: Resources and Environment Division, Ministry of Marine Resources, 1995.

Satapoomin, U., and K. Chawanon. “The Small-Scale Reef Fishery at Phuket Island, Thailand Andman Sea Coast.” *Ten Years after Bleaching: Facing the Consequences of Climate Change in the Indian Ocean (CORDIO Status Report 2008)*, 2008, 285–91.

Secretariat of the Pacific Community. “Fish and Food Security.” Policy Brief. New Caledonia: Secretariat of the Pacific Community, 2008.

Sobolevskaya, A., and E. Divovich. “The Wall Street of Fisheries: The Russian Far East, a Catch Reconstruction from 1950 to 2010.” Working Paper Series. Vancouver: Fisheries Centre, The University of British Columbia, 2015.

Tesfai, W. “Seafood Consumption in the Dahlak Archipelago, Eritrea.” Personal communication. Toronto: York University, 2015.

van Beukering, P., W. Haider, M. Longland, H. Cesar, J. Sablan, S. Shjegstad, B. Beardmore, Y. Liu, and G. Omega Garces. “The Economic Value of Guam’s Coral Reefs.” University of Guam Marine Laboratory Technical Report. Mangilao, Guam: University of Guam, 2007.

Zylich, K., S. Harper, R. Licandeo, R. Vega, D. Zeller, and D. Pauly. “Fishing in Easter Island, a Recent History (1950 2010).” *Latin American Journal of Aquatic Research* 42, no. 4 (October 10, 2014): 845–56.
