# Supplementary material for: A Global Estimate of Seafood Consumption by Coastal Indigenous Peoples
Source: PLoS One. 2016 Dec 5;11(12):e0166681. doi: 10.1371/journal.pone.0166681 (PMC5137875; doi:10.1371/journal.pone.0166681)
Supplement: S2 Text — (DOCX) [file pone.0166681.s006.docx]

**S2 Text. Sensitivity analysis methods and implications for seafood consumption estimates.**

In order to test the sensitivity of consumption estimates to starting data, we performed a jackknife analysis running the meta-analytical model 5,000 times omitting a random subset of total observed (reference) data points on each iteration. Figure 4 (in manuscript) shows subsequent total global consumption estimates, with 10% of data points omitted at random before each model run. The range of consumption estimates given variable data input is narrow (coefficient of variation= 3.6%), with a tendency to underestimate consumption relative to the baseline estimate using all available data.

We also tested the sensitivity of the above jackknifing method to the percentage of initial data points excluded from the model on each iteration (i.e. the baseline 10% value used above). As expected, the data exclusion ratio was directly correlated with the coefficient of variation (CV) of estimated total consumption (S1 Fig) (i.e. model outputs become more uncertain as less data are available). Notably, however, consumption estimates were negatively correlated with the data exclusion ratio (S1 Fig). This indicates that the model tends to underestimate consumption when data are not available, reflecting our systematic conservative assumptions throughout the model framework.

This result is supported by testing accuracy of individual data point estimates. In this case, one data point at a time is excluded from the model and the estimated consumption per capita for that data point is then compared with the observed (reference) value. Results show underestimation of consumption for higher observed values and, importantly, no unreasonable consumption estimates (S2 Fig).

After accounting for potential variability due to initial data points, the CV of total global consumption estimates is 3.6%. At the regional and subregional level, coefficients range between 4—14% (S6 Table). The Americas and Oceania regions are most robust to initial data availability, with relatively higher uncertainty in estimates for Africa and Europe (S2 Table).
